# Supplementary material for: Amyotrophic lateral sclerosis: Correlations between fluid biomarkers of NfL, TDP-43, and tau, and clinical characteristics
Source: PLoS One. 2021 Nov 29;16(11):e0260323. doi: 10.1371/journal.pone.0260323 (PMC8629269; doi:10.1371/journal.pone.0260323)
Supplement: S1 Table — (DOCX) [file pone.0260323.s001.docx]

**Supplementary Table 1:** Clinical information and concentrations of biomarkers.

| Case | Sex | Age (years) | Diagnosis | UMN score | MRC sum score | %VC (%) | ALSFRS-R | Progression rate (/month) | Split hand index | Corrected levels in CSF (pg/mL) | | |  | Corrected levels in plasma (pg/mL) | | |
| --- | --- | --- | --- | --- | --- | --- | --- | --- | --- | --- | --- | --- | --- | --- | --- | --- |
|  |  |  |  |  |  |  |  |  |  | NfL | TDP-43 | t-tau |  | NfL | TDP-43 | t-tau |
| 1 | F | 59 | Definite | 13 | 12 | 36 | 18 | 0.67 | N/A | 5018 | 68 | 11 |  | 41 | 237 | 0.67 |
| 2 | M | 75 | Probable | 12 | 52 | 45 | 34 | 0.70 | N/A | 14408 | 64 | 13 |  | 209 | 78 | 0.72 |
| 3 | F | 60 | Probable | 5 | 50 | 133 | 45 | 0.30 | N/A | 4884 | 64 | 15 |  | 34 | 284 | 0.50 |
| 4 | M | 73 | Possible | 2 | 51 | 104 | 34 | 1.56 | N/A | 1264 | 61 | 5 |  | 20 | 146 | 0.46 |
| 5 | M | 65 | Possible | 2 | 34 | 110 | 35 | 1.30 | N/A | 15971 | 62 | 12 |  | 83 | 268 | 1.10 |
| 6 | M | 57 | Definite | 5 | 42 | N/A | 24 | 0.69 | N/A | 11126 | 62 | 6 |  | 68 | 28 | 0.47 |
| 7 | M | 79 | Probable | 10 | 46 | 70 | 32 | 0.24 | N/A | 5116 | 59 | 13 |  | 123 | 103 | 0.36 |
| 8 | M | 65 | Probable | 4 | 49 | 114 | 45 | 0.10 | N/A | 10081 | 66 | 18 |  | 101 | 562 | 0.17 |
| 9 | M | 56 | Possible | 9 | 59 | 115 | 46 | 0.33 | N/A | 1052 | 60 | 7 |  | 16 | 1380 | 0.40 |
| 10 | F | 62 | Probable | 8 | 56 | 122 | 46 | 0.13 | N/A | 1706 | 60 | 12 |  | 18 | 636 | 0.29 |
| 11 | F | 74 | Possible | 12 | 60 | 124 | 47 | 0.13 | 21.0 | 7122 | 62 | 18 |  | 79 | 243 | 0.85 |
| 12 | M | 62 | Probable | 3 | 56 | 112 | 44 | 0.80 | 0.6 | 18872 | 61 | 16 |  | 84 | 1167 | 0.34 |
| 13 | F | 72 | Probable | 9 | 53 | 75 | 36 | 0.92 | 4.8 | 17506 | 65 | 17 |  | 313 | 774 | 0.86 |
| 14 | M | 63 | Probable | 8 | 59 | 65 | 37 | 1.00 | N/A | 17319 | 59 | 13 |  | 204 | 260 | 0.87 |
| 15 | F | 85 | Possible | 3 | 55 | 116 | 45 | 0.10 | 12.3 | 3455 | 57 | 22 |  | 46 | 595 | 1.05 |
| 16 | M | 74 | Probable | 10 | 54 | 88 | 40 | 1.14 | N/A | 15092 | 64 | 24 |  | 144 | 384 | 2.60 |
| 17 | M | 38 | Possible | 5 | 56 | 103 | 43 | 0.15 | 11.9 | 879 | 67 | 10 |  | 9 | 1037 | 0.33 |
| 18 | M | 81 | Probable | 5 | 50 | 110 | 41 | 0.78 | 11.9 | 18322 | 58 | 16 |  | 228 | 525 | 0.54 |
| 19 | F | 43 | Definite | 13 | 60 | 91 | 46 | 0.25 | 6.8 | 14403 | 61 | 9 |  | 115 | 143 | 0.73 |
| 20 | F | 71 | Suspected | 0 | 51 | 74 | 44 | 0.24 | 6.5 | 3386 | 61 | 19 |  | 68 | 763 | 0.16 |
| 21 | M | 75 | Definite | 5 | 38 | N/A | 30 | 2.00 | 9.7 | 15856 | 67 | 11 |  | 401 | 1113 | 0.42 |
| 22 | F | 59 | Probable | 3 | 36 | 85 | 39 | 0.16 | N/A | 3376 | 66 | 18 |  | 33 | 154 | 0.23 |
| 23 | M | 72 | Possible | 10 | 60 | 94 | 40 | 0.14 | 14.3 | 1520 | 66 | 2 |  | 29 | 176 | 0.96 |
| 24 | M | 53 | Possible | 10 | 58 | 124 | 48 | 0.00 | 5.0 | 11900 | 64 | 11 |  | 84 | 4620 | 1.02 |
| 25 | F | 76 | Definite | 9 | 54 | 89 | 39 | 0.90 | 2.1 | 6148 | 63 | 14 |  | 67 | 3528 | 0.46 |
| 26 | M | 60 | Definite | 12 | 47 | 67 | 32 | 0.41 | 8.9 | 13942 | 63 | 10 |  | 85 | 3000 | 0.40 |
| 27 | F | 66 | Probable | 13 | 45 | 83 | 40 | 1.14 | 0.5 | 23720 | 63 | 13 |  | 147 | 676 | 0.61 |
| 28 | M | 38 | Probable | 12 | 57 | 87 | 47 | 0.14 | 5.7 | 7984 | 60 | 10 |  | 59 | 442 | 0.60 |
| 29 | M | 84 | Definite | 16 | 55 | N/A | 33 | 3.00 | 2.3 | 21485 | 69 | 13 |  | 390 | 404 | 0.70 |
| 30 | F | 75 | Definite | 7 | 45 | 94 | 37 | 2.20 | 8.6 | 28477 | 40 | 24 |  | N/A | N/A | N/A |
| 31 | M | 79 | Definite | 5 | 58 | 97 | 42 | 0.38 | 3.2 | 3421 | 55 | 15 |  | N/A | N/A | N/A |
| 32 | M | 56 | Suspected | 0 | 54 | 107 | 42 | 1.00 | 10.6 | 14708 | 53 | 7 |  | N/A | N/A | N/A |
| 33 | F | 65 | Possible | 2 | 55 | 107 | 46 | 0.11 | 19.3 | 3211 | 87 | 21 |  | N/A | N/A | N/A |
| 34 | F | 65 | Suspected | 0 | 55 | 71 | 42 | 0.43 | 10.0 | 3711 | 72 | 11 |  | N/A | N/A | N/A |
| 35 | F | 81 | Probable | 7 | 60 | 70 | 41 | 0.54 | 5.8 | 4821 | 66 | 11 |  | N/A | N/A | N/A |
| 36 | M | 51 | Possible | 5 | 60 | 94 | 44 | 0.10 | 0.9 | 4305 | 70 | 11 |  | N/A | N/A | N/A |
| 37 | M | 78 | Probable | 1 | 47 | 106 | 43 | 0.63 | 1.6 | 3465 | 57 | 12 |  | N/A | N/A | N/A |
| 38 | M | 74 | Possible | 1 | 57 | 110 | 42 | 0.09 | 11.6 | 4202 | 95 | 9 |  | N/A | N/A | N/A |
| 39 | F | 71 | Suspected | 0 | 51 | 74 | 41 | 0.41 | 3.5 | 3485 | 75 | 18 |  | N/A | N/A | N/A |
| 40 | M | 77 | Definite | 3 | 49 | 84 | 30 | 1.64 | 5.7 | 8048 | 70 | 11 |  | N/A | N/A | N/A |
| 41 | F | 75 | Possible | 15 | 58 | 69 | 41 | 2.33 | 4.2 | 4971 | 55 | 23 |  | N/A | N/A | N/A |
| 42 | F | 77 | Definite | 12 | 60 | 39 | 35 | 0.93 | 9.8 | 10366 | 73 | 22 |  | N/A | N/A | N/A |
| 43 | F | 76 | Definite | 11 | 39 | 89 | 20 | 2.00 | 0.2 | 11908 | 76 | 29 |  | N/A | N/A | N/A |
| 44 | F | 66 | Definite | 11 | 58 | 86 | 46 | 0.29 | 8.7 | 3175 | 104 | 12 |  | N/A | N/A | N/A |
| 45 | M | 78 | Definite | 8 | 47 | N/A | 26 | 0.50 | 1.5 | 3260 | 97 | N/A |  | N/A | N/A | N/A |
| 46 | F | 77 | Definite | 12 | 56 | 89 | 39 | 1.00 | 0.6 | 6980 | 73 | 11 |  | N/A | N/A | N/A |
| 47 | M | 69 | Possible | 1 | 50 | 84 | 43 | 0.25 | 12.9 | 7947 | 33 | 11 |  | N/A | N/A | N/A |
| 48 | F | 66 | Possible | 13 | 45 | 83 | 40 | 1.14 | 0.5 | 21967 | 64 | 16 |  | 155 | 786 | 0.32 |
| 49 | F | 78 | Possible | 2 | 58 | 93 | 47 | 0.13 | 1.3 | 4824 | 81 | 15 |  | N/A | N/A | N/A |
| 50 | M | 73 | Definite | 9 | 52 | 99 | 42 | 0.86 | 13.9 | 2105 | 87 | 11 |  | N/A | N/A | N/A |
| 51 | M | 37 | Probable | 12 | 57 | 87 | 47 | 0.14 | 5.7 | 11290 | 61 | 11 |  | 66 | 1529 | 0.74 |
| 52 | M | 77 | Probable | 10 | 44 | 47 | 36 | 1.33 | 0.8 | N/A | N/A | N/A |  | 129 | 471 | 0.51 |
| 53 | F | 85 | Probable | 10 | 32 | 44 | 25 | 1.28 | 4.9 | 9223 | 73 | 11 |  | N/A | N/A | N/A |
| 54 | F | 72 | Definite | 9 | 50 | 62 | 40 | 0.26 | 6.5 | 7342 | 68 | 15 |  | 109 | 473 | 1.01 |
| 55 | M | 75 | Definite | 5 | 55 | N/A | 16 | 5.33 | 0.2 | N/A | N/A | N/A |  | 68 | 313 | 0.32 |
| 56 | M | 84 | Definite | 16 | 55 | N/A | 33 | 0.12 | 2.3 | 22292 | 53 | 13 |  | 378 | 410 | 0.73 |
| 57 | M | 73 | Definite | 9 | 46 | 66 | 37 | 0.73 | 1.5 | 8081 | 61 | 10 |  | 342 | 267 | 1.41 |
| 58 | M | 68 | Definite | 13 | 50 | 84 | 40 | 0.35 | 2.7 | 9368 | 94 | 12 |  | N/A | N/A | N/A |
| 59 | F | 67 | Probable | 12 | 58 | 80 | 43 | 0.24 | 2.2 | 6980 | 58 | 14 |  | N/A | N/A | N/A |
| 60 | M | 76 | Suspected | 0 | 52 | 104 | 41 | 0.47 | 11.3 | 4394 | 62 | 19 |  | N/A | N/A | N/A |
| 61 | M | 53 | Probable | 6 | 49 | 72 | 42 | 0.50 | 3.7 | 10567 | 67 | 7 |  | 180 | 135 | N/A |
| 62 | M | 70 | Definite | 16 | 35 | 73 | 31 | 0.68 | 4.4 | 11814 | 55 | 7 |  | 249 | 391 | 0.63 |
| 63 | M | 76 | Probable | 6 | 49 | 83 | 38 | 0.45 | 18.3 | 9918 | 66 | 18 |  | N/A | N/A | N/A |
| 64 | M | 63 | Probable | 6 | 58 | 114 | 47 | 0.05 | 3.4 | 10691 | 61 | 11 |  | 129 | 861 | 0.63 |
| 65 | M | 70 | Definite | 5 | 55 | 27 | 26 | 1.69 | 5.1 | 6942 | 60 | 11 |  | 148 | 138 | 1.22 |
| 66 | M | 81 | Definite | 10 | 56 | 84 | 34 | 1.17 | 2.2 | 21648 | 49 | 12 |  | 230 | 761 | 0.76 |
| 67 | M | 69 | Probable | 4 | 38 | 56 | 31 | 2.83 | 1.9 | N/A | N/A | N/A |  | 230 | 184 | 0.85 |
| 68 | F | 83 | Probable | 6 | 41 | 64 | 36 | 0.75 | 2.0 | 4962 | 63 | 11 |  | 124 | 198 | 1.04 |
| 69 | M | 79 | Suspected | 0 | 52 | 73 | 42 | 0.14 | 0.9 | 4246 | 72 | 13 |  | N/A | N/A | N/A |
| 70 | M | 62 | Definite | 16 | 57 | 91 | 47 | 0.14 | 7.0 | 11371 | 59 | 8 |  | 131 | 386 | 0.40 |
| 71 | M | 76 | Suspected | 9 | 60 | 91 | 46 | 0.08 | 12.9 | 5755 | 61 | 14 |  | 97 | 748 | 4.07 |
| 72 | M | 71 | Probable | 3 | 55 | 82 | 46 | 0.07 | 2.0 | 14456 | 56 | 8 |  | 155 | 236 | 0.17 |
| 73 | M | 78 | Probable | 1 | 46 | 90 | 43 | 0.20 | 12.6 | 8373 | 59 | 14 |  | 160 | 68 | 0.74 |
| 74 | F | 60 | Probable | 10 | 51 | 98 | 43 | 1.00 | 14.1 | N/A | N/A | N/A |  | 100 | 215 | 1.06 |
| 75 | M | 71 | Probable | 9 | 43 | 79 | 37 | 0.35 | 2.3 | N/A | N/A | N/A |  | 90 | 258 | 0.17 |
|  | M:F 47 : 28 | Median [IQR] 72 [63 - 77] | Definite:  N=25 Probable:  N=28 Possible:  N=15 Suspected:  N=7 | Median [IQR] 8 [4 - 11] | Median [IQR] 52 [47 - 57] | Median [IQR] 87 [73 - 103] | Median [IQR] 41 [35 - 44] | Median [IQR] 0.47 [0.16 - 1.00] | Median [IQR] 5.0 [2.0 - 10.0] | Median [IQR] 7965 [4261 - 13433] | Median [IQR] 63 [60 - 68] | Median [IQR] 12 [11 - 16] |  | Median [IQR] 109 [68 - 160] | Median [IQR] 391 [215 - 761] | Median [IQR] 0.63 [0.40 - 0.86] |
| UMN, upper motor neuron; MRC, Medical Research Council; VC, vital capacity; ALSFRS-R, amyotrophic lateral sclerosis functional rating scale revised score; CSF, cerebrospinal fluid; | | | | | | | | | | | | | | | | |
| NfL, neurofilament light chain; TDP-43, TAR DNA-binding protein 43; t-tau, total tau; N/A, not applicable | | | | | | | | | | | | | | | | |
| This cohort included four familial ALS cases: cases 3 (younger brother), 8 (maternal grandmother), 41 (younger brother), and 57 (mother) (gray shading). No genetic abnormality was identified in this family. | | | | | | | | | | | | | | | | |
