# Supplementary material for: Amyotrophic lateral sclerosis: Correlations between fluid biomarkers of NfL, TDP-43, and tau, and clinical characteristics
Source: PLoS One. 2021 Nov 29;16(11):e0260323. doi: 10.1371/journal.pone.0260323 (PMC8629269; doi:10.1371/journal.pone.0260323)
Supplement: Attachment — Submitted filename: Point-by-point responce20211002.docx [file pone.0260323.s002.docx]

**Responses to the reviewers’ comments**

We appreciate the thoughtful comments from the reviewers that helped us to improve this manuscript. We have revised the manuscript as suggested, and believe that it has been significantly improved.

**Reviewer #1**
This research is interesting, but there might be some errors and main concerns:
In P3, There might be an error. “Plasma TDP-43 correlated negatively with split hand index......” was shown in “Results”, but in “Conclusions”, it was shown “The positive correlation between plasma TDP-43 and split hand index......”.

**Response:**

We apologize for our careless mistakes. We have revised the sentence in Conclusions of Abstract and Conclusion:

" The negative correlation between plasma TDP-43 and split hand index may support the pathophysiological association between plasma TDP-43 and ALS." (Page 3, lines 57 - 58)

“A negative association between TDP-43 in plasma and split hand index was identified.” (Page 17, lines 356-357)

This study cohort consisted of 75 ALS patients, it might be better for the authors to indicate the patients are only sporadic or sporadic and familial cases.

**Response:**

Thank you for your suggestion. We have added a sentence to Results:

" This cohort included four familial ALS cases in which relatives with ALS or motor neuron disease were found within the second degree by consanguinity: cases 3 (younger brother), 8 (maternal grandmother), 41 (younger brother), and 57 (mother) (S Table). In those familial cases, no genetic abnormality was identified." (Page 9, lines 174 - 178)

In P6, The authors indicated that “The participating ALS patients were diagnosed as suspected, possible, probable, or definite ALS based on revised El Escorial criteria.” . But in revised El Escorial criteria, there are four diagnostic categories, clinically definite, clinically probable, clinically probable–laboratory-supported and clinically possible. The category of “clinically suspected ALS was deleted from the revised El Escorial Criteria for the Diagnosis of ALS.

**Response:**

Thank you for your comment about the diagnostic criteria applied in this study. We included cases with progressive muscle atrophy as suspected ALS. As the reviewer commented, there is no category of suspected ALS in revised El Escorial criteria, and so we changed "revised El Escorial criteria" to "El Escorial criteria":

" The participating ALS patients were diagnosed with suspected, possible, probable, or definite ALS based on El Escorial criteria (12)." (Page 6, lines 90 - 95)

The authors used “% vital capacity” as a clinical data, did it mean “% forced vital capacity (FVC)”? And, if ALS patients had bulbar dysfunction, decreased % FVC could not reflect the real situation of respiratory function. Even if all participants had normal bulbar function, it might be better to analyze the relation of abnormal % FVC with those fluid biomarkers.

**Response:**

Thank you for the comment. In our study, "% vital capacity" meant "slow vital capacity", not FVC. We considered that bulbar dysfunction less affected the value of % slow VC compared with % forced VC. To avoid misunderstanding, we have clarified this point in Methods, as follows:

“At the time of CSF collection, clinical data including age, sex, upper motor neuron (UMN) score, Medical Research Council (MRC) sum score, % slow vital capacity (%VC), ALS Functional Rating Scale revised (ALSFRS-R)” (Page 6, lines 97-100)

“progression rate” was used in this study, it might be better to use “disease progression rate (DPR)”.

**Response:**

Following the reviewer's suggestion, we have replaced progression rate with DPR.

“split hand index” was used as a clinical data in this study, but the hand muscles innervated by median and ulnar nerves might not be involved in some patients in this cohort. And it was shown “the split hand index was calculated by dividing the product of the compound muscle action potential (CMAP) amplitude recorded over the first dorsal interosseous and abductor pollicis brevis by the CMAP amplitude recorded over the abductor digiti minimi ” in page 7, but the authors did not indicate that nerve conduction study was made in which side, left or right, or the involved side. These will certainly influence the research results.

**Response:**

We agree with the reviewer's comment on the appropriateness of using split hand index in this study. We considered that split hand index sensitively reflected the pathophysiology of ALS because it is a specific phenomenon in ALS and useful for its early diagnosis. These are why we applied split hand index as an electrophysiological parameter for ALS in this study. Following the reviewer's comment, we have indicated the side subjected to NCS:

" Based on the nerve conduction study, split hand index was calculated by dividing the product of the compound muscle action potential (CMAP) amplitude recorded over the first dorsal interosseous and abductor pollicis brevis by the CMAP amplitude recorded over the abductor digiti minimi on the affected side (17)." (Page 7, lines 112- 118)

In discussion, it might be better to discuss the probable reasons of the most important research results in more detail.

**Response:**

We appreciate this comment. We actually consider the most important findings to be the negative correlation between NfL and TDP-43 in CSF and association between CSF TDP43 and slow progression of the disease, which suggest that CSF TDP-43 elevation in ALS is not a simple consequence of its release into CSF during neurodegeneration. We have described the postulated mechanism for the phenomenon on Page 14, lines 276-287. However, we also acknowledge that the insufficient fitness of the multivariate analysis regarding CSF TDP-43 might hamper such direct interpretation. Therefore, we would like to refrain from overemphasizing our hypothetical idea, which might be too speculative for some readers.

**Reviewer #2**

This is an elegant study on a fundamental topic in ALS biomarker research. The authors are experts in the field. The results are interesting and well presented. I have only some minor issues:
1. Line 91: the authors state that the patients of the discovery and replication cohorts were "extracted" from the cohorts of a previous study. It is not clear whether only some of the patients evaluated in the previous study were taken, and, if so, which criteria were applied for this selection.

**Response:**

We thank the reviewer for the important comment. We did not select some patients enrolled in our previous study, and used whole data. We apologize for the misleading expression and have revised the sentence:

" The study cohort consisted of 75 ALS patients visiting our institute between September 2009 and May 2018, who were from the discovery cohort enrolled from September 2009 to March 2014 (n=29) and validation cohort enrolled from April 2014 to May 2018 (n=46) included in our previous study (11)." (Page 6, lines 90 - 95)

2. Lines 134-136 ("All samples were analyzed in duplicate on one occasion. For this sub-analysis, we used the levels of each biomarker in the ALS group of our previous paper"). I think that these sentences are not so clear. Does this mean that for these patients the levels of the biomarkers were not measured again after the work made for the previous publication? Please clarify.

**Response:**

We apologize for the unclear description. We did not measure each biomarker level again after the previous publication and used the same raw data as in our previous study. Because the inter-assay variation between the discovery and validation cohorts was not negligible, we corrected the values using internal controls in the previous study. In the current study, we used those conbined biomarkers for the analyses. We added the following description:

"All samples were analyzed in duplicate on one occasion independently in the two cohorts (discovery and validation cohorts). Because of inter-assay variation between the two cohorts, we corrected the values based on those of internal controls (11). For this sub-analysis in the present study, we used the same levels of those combined values of each biomarker in the ALS group of our previous paper." (Page 8, lines 135 - 140)

3. Line 190: "genialized" --> "generalized"?

**Response:**

We apologize for our mistake. We have corrected the word.

4. Lines 191-194 ("we would like to emphasize that CSF TDP-43 were negatively associated with the progression rate both in univariate and multivariate analyses regardless of significance, in contrast to those in CSF NfL and CSF tau"). I do not think that is correct to equalize a significant result (multivariate analysis) and a non-significant one. Perhaps the above-mentioned sentence could be eliminated.

**Response:**

We appreciate the reviewer's instructive comment, and have deleted the sentence.

5. Lines 353-354. Here it is written that the association between plasma TDP-43 and the split hand index is positive. However, I think that it is negative.

**Response:**

We would like to apologize for our careless mistakes. We have corrected the relevant part.

We have revised the sentence in Conclusions of Abstract and Conclusion:

" The negative correlation between plasma TDP-43 and split hand index may support the pathophysiological association between plasma TDP-43 and ALS." (Page 3, lines 57 - 58)

“A negative association between TDP-43 in plasma and split hand index was identified.” (Page 17, lines 356-357)

6. In table 1 I see that median t-Tau in CSF in ALS patients was 12 pg/mL. It seems a low value. Do the authors confirm this value?

**Response:**

We thank the reviewer for this insightful comment. As the reviewer mentioned, the median t-tau value was lower than reported. This discrepancy may be due to insufficient inter-assay concordance of Simoa kits, being markedly inferior than reported by the Simoa supplier. As described, we measured the biomarker values in the two independent cohorts (discovery and validation), but there were non-negligible mean-value differences between the two. So, we combined the data by correction calculation based on the data of internal controls. In the case of t-tau, the validation cohort data were adjusted to lower values than expected as a result of correction calculation, because the measurements of another cohort were relatively low. Those technical issues were described in Fig. 6 and Supplementary Table 3 of the previous study. To clarify those points, we have added the following description to Methods:

"All samples were analyzed in duplicate on one occasion independently in the two cohorts (discovery and validation cohorts). Because of inter-assay variation between the two cohorts, we corrected the values based on those of internal controls (11). For this sub-analysis in the present study, we used the same levels of those combined values of each biomarker in the ALS group of our previous paper." (Page 8, lines 135 - 140)

**Reviewer #3**

The authors extended their analysis of CSF NfL and TDP-43 from a cohort of patients with ALS (published previously), by comparing measures to commonly used clinical measures of disease progression. Their methodology is clearly described and I have no concerns with their approach. I have no comments on their approach. This is sound. I have a few minor suggestions and some points of interest.
1. I initially suspected that the correlation between CSF NfL and TDP43 was driven by a few outliers. However after closer consideration (thank you for providing data points for individual patients) it appears that 5 of the participants present with very low CSF NfL levels relative to their TDP43 scores (patients 4, 9, 10, 17 and 23). Most of these individuals have very slow progression rates, except for #4, which has a very fast progression rate. Did the authors note any specific clinical features in these patients that may explain these low NfL scores. Could other clinical features be relevant? I note that all of these patients had a possible/probable diagnosis at study inclusion.

**Response:**

We are grateful for the important suggestion. As indicated, those cases (4, 9, 10, 17, and 23) with low NfL and relative TDP43 generally showed a low progression rate and retained diagnoses of possible or probable at enrollment. We have reviewed their clinical charts and found that those cases showed lower motor neuron predominant symptoms at enrollment, (but their symptoms eventually meet the criteria of probable or definite ALS). These findings correspond to the knowledge that elevation of CSF NfL reflects neurodegeneration per unit-time and that such elevation is associated with degeneration of upper rather than that of lower motor neurons in general (Gaiani A, et al. JAMA Neurol 74. 525.2017, ref. 5). However, we could not put forward any good suggestions “why they had relatively high CSF TDP43 values” based on their clinical records.

2. Did familial/sporadic status impact the results? Is the genetic status of patients known? If so, can the authors please comment.

**Response:**

Thank you for your constructive suggestion. There were only four cases involving a relative with motor neuron disease within the second degree and genetic abnormalities were not identified in any familial cases. Following the reviewer's suggestion, we have added this point to Results:

" This cohort included four familial ALS cases in which relatives with ALS or motor neuron disease were found within the second degree by consanguinity: cases 3 (younger brother), 8 (maternal grandmother), 41 (younger brother), and 57 (mother) (S Table). In those familial cases, no genetic abnormality was identified." (Page 9, lines 174 - 178)

3. I caution the authors against the use of “biomarker” for their current data results; factors are tested as possible prognostic indicators. This limitation is noted in the discussion, however the authors are asked to revise the term “biomarker” in their abstract.

**Response:**

Thank you for your comment on the use of "biomarker". We replaced the term "biomarker" with "marker" in some parts of Abstract.

4. Figure legend 1: Please correct the figure legend to state that correlations between plasma and CSF markers WERE completed in the previous manuscript. This should be followed by a reference to the report.

**Response:**

Thank you for your suggestion; we revised the Figure 1 legend as follows:

" Correlations between the same markers in CSF and plasma were presented in our previous report (11)." (Page 12, lines 236 - 237)

5. The manuscript is well-written and easy to follow, however I’ve noted a few minor grammatical mistakes; i.e. line 308 “missing the word “is” between CSF and elevated. The authors are encouraged to revise the document.

**Response:**

We would like to thank you for the comment. We have revised the manuscript as well as asked an English proofreader to check for grammatical errors in the final revised manuscript. His corrections were shown as Track-change of the Word. We attached his certification of proofing below.
